# Development of Supercooling Preservation Method for Adherently Cultured Endothelial Cells and Its Application to Microphysiological Systems

**DOI:** 10.3390/cells15070619

**Published:** 2026-03-30

**Authors:** Maaya Hikichi, Tsutomu Shimoda, Kiichi Sato

**Affiliations:** 1 School of Science and Technology, Gunma University, Tenjin-cho, Kiryu 376-8515, Gunma, Japan; 2 R&D Division, Sanden Retail Systems Corporation, ARCA West 8F, 1-2-4 Kinshi, Sumida-ku, Tokyo 130-8563, Japan

**Keywords:** adherent cells, cell preservation, HepG2, TMNK-1, microphysiological systems, organ-on-a-chip, supercooling preservation

## Abstract

Microphysiological systems (MPS) that recapitulate human organ functions have gained attention as alternatives to animal experiments in drug discovery, regenerative medicine, and toxicity assessments. However, preserving MPS with adherent cells remains a significant challenge. In this study, we developed a supercooling preservation method that enables the low-temperature storage of human-derived adherent cells without freezing. Using human hepatic sinusoidal endothelial cells (TMNK-1), we optimized the preservation conditions by assessing the temperature, cooling and rewarming rates, and preservation solutions. Under optimized conditions (preservation at −4 °C, −0.028 °C/min cooling, and +1.0 °C/min rewarming), high cell viability and preserved morphology were maintained for up to 7 days. When these conditions were applied to both two- and three-dimensional MPS containing TMNK-1 or HepG2 cells, post-preservation viability remained high, and no cell death or cytoskeletal disruption was observed. This supercooling preservation method has the potential to serve as a practical strategy for the temporary storage of MPS.

## 1. Introduction

In drug discovery, regenerative medicine, and toxicity assessment, there is an increasing demand for alternatives to animal experiments. The use of microphysiological systems (MPS) that mimic human organ function and microenvironments has been rapidly expanding. MPS consists of cells adherently cultured within microscale channels, and the medium flow recreates an in vivo environment. This enables a more precise prediction of pharmacokinetics and toxicity, making MPS a high-precision assessment platform without individual animals. Organ models, including the liver [1,2,3], kidney [4,5], lung [6], and vasculature [7], have been developed as MPS, with potential applications in disease modeling and personalized medicine.

However, a major limitation for their widespread use is the lack of reliable methods for preservation. Currently, numerous MPS-based experiments assume that cell seeding, culture, and drug testing occur in the same facility over a short period, and no established method exists to stably preserve MPS containing adherent cells. This represents a major bottleneck for the dissemination and broader use of MPS.

Cryopreservation is the most widely used method for long-term storage of cells, enabling preservation for years by cooling to ultra-low temperatures (−80 to −196 °C) [8,9]. However, it is primarily designed for cell suspension and is challenging to apply to adherent cells. During storage, ice crystal formation can mechanically damage cell membranes and intracellular structures [10], and osmotic stress generated during cooling and warming can induce cell death [11]. Additionally, cryopreservation often uses solutions containing cryoprotective agents—such as dimethyl sulfoxide (DMSO) [12,13]—that are cytotoxic and pose practical limitations. Furthermore, MPS often possess intricate architectures, such as multilayer structures composed of different materials and cells, making them susceptible to structural damage during freezing. Therefore, cryopreservation of adherent cell-containing MPS is challenging.

Against this background, supercooling preservation has recently gained attention. This method preserves cells at subzero temperatures (approximately −20 to 0 °C) without ice crystal formation, thereby minimizing metabolic activities and extending the preservation period [14]. Under supercooling conditions, freezing is avoided [15,16,17], oxygen consumption and energy metabolism are reduced [18], and cellular stress is minimized [19]. Preservation of suspended rat hepatocytes for 7 days at −4 °C has been reported [18]. In particular, recent studies have reported the successful supercooling preservation of adherent monolayer cultures, such as primary rat hepatocytes cultured on well plates, with viability maintained for up to 2 days at −2 °C [19].

In our previous study, we developed a supercooling preservation method that maintains adherently cultured human hepatoma cells (HepG2) in a well plate for up to 14 days at −4 °C [20]. HypoThermosol FRS was used as the preservation solution, and the cooling and rewarming rates were controlled at −0.028 °C/min and +1.0 °C/min, respectively, to achieve high post-preservation cell viability. The preserved cells maintained normal morphology, demonstrated no signs of apoptosis or cell death, and were immediately usable after rewarming. This represents a significant advantage over conventional cryopreservation, greatly enhancing the flexibility and efficiency of cell-based experiments.

This supercooling preservation approach offers promise for mid-term preservation of adherent cells while avoiding cryopreservation-associated limitations. However, the aforementioned study focused exclusively on a single epithelial cell line (HepG2) cultured in a two-dimensional (2D) well plate, without demonstrating its applicability to other cell types, specifically those with distinct morphologies and functions. Moreover, no studies have addressed the application of this method to microfluidic devices with complex architectures, such as MPS.

In this study, immortalized human liver-derived endothelial cells were used to evaluate the applicability of the supercooling preservation method as a cell type distinct from the hepatocyte cell line. Furthermore, we expanded this methodology to more complex systems by applying the supercooling preservation technique to MPS. The objective of this study was a preliminary investigation of the preservation conditions for these cells, and the cell viability and morphological changes after preservation were examined.

## 2. Materials and Methods

### 2.1. Experimental Flow

**Figure 1:**
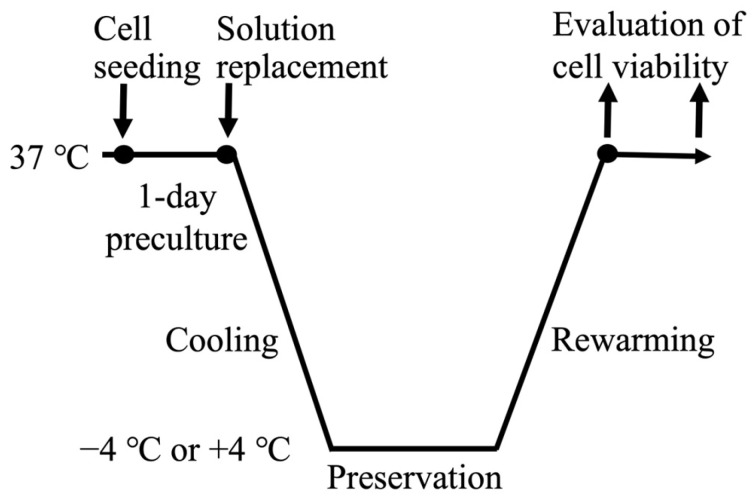
Outline of the preservation experiment procedure.

Figure 1 summarizes the experimental procedure. After 24 h of preculture, the culture medium was exchanged for either fresh medium or one of two commercially available preservation solutions. The following fluids were used in the experiment: HypoThermosol FRS (FRS; BioLife Solutions, Bothell, WA, USA) and Thelio Keep (TK; BioVerde, Kyoto, Japan). The cells were then cooled to either −4 °C or +4 °C at a defined rate in a temperature-controlled CO_2_ incubator MGI-150X-C (Sanden Retail Systems, Tokyo, Japan). Once the target temperature had been reached, the cells were held at −4 °C or +4 °C in a humidified 5% CO_2_ atmosphere and subsequently rewarmed to 37 °C at a constant rate. Temperatures lower than −4 °C were excluded because of the risk of ice formation. In the immediate-cooling experiments, cells maintained at 37 °C were transferred directly to an incubator preadjusted to −4 °C or +4 °C. In the immediate-rewarming experiments, preserved cells were moved directly into a CO_2_ incubator set at 37 °C. For non-preserved controls, cells were cultured at 37 °C for 24 h in 96-well plates or microfluidic devices. To examine the short- and mid-term preservation performance of adherent cells and MPS, preservation periods of 1 and 2 days and 5, 7, and 14 days were selected. These intervals were intended to track progressive changes in cell viability over time and to evaluate how stably the cells could be maintained during preservation. Thus, the chosen time points enabled efficient assessment of temporal viability trends.

### 2.2. Fabrication of Microfluidic Devices

#### 2.2.1. Microdevice for 2D Culture

**Figure 2:**
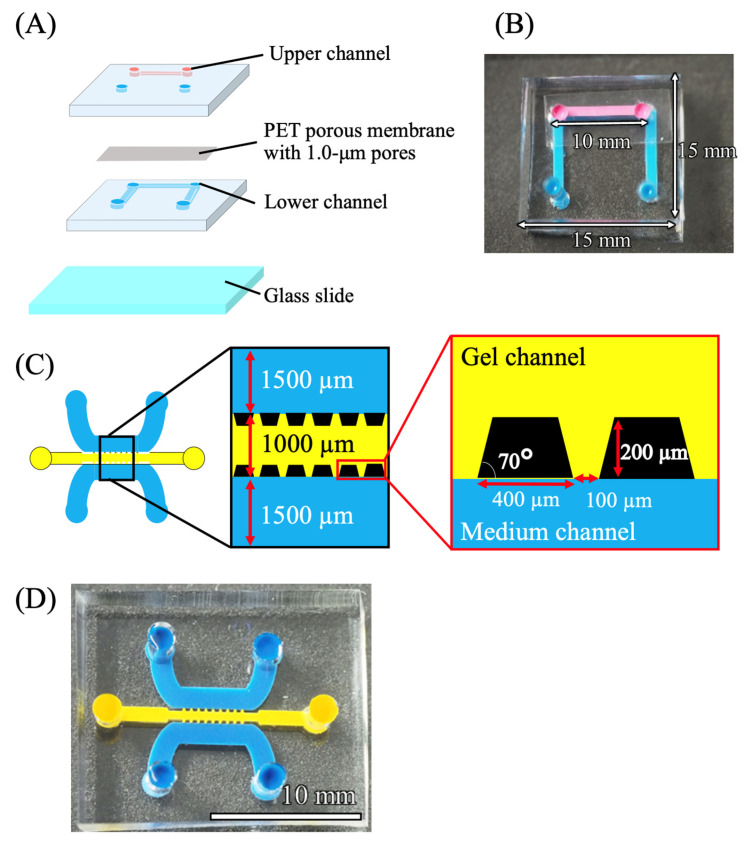
Microfluidic devices for microphysiological systems. (**A**) Schematic diagram and (**B**) photograph of the device used for two-dimensional culture. (**C**) Schematic diagram and (**D**) photograph of the device used for three-dimensional culture.

The 2D culture microdevice consisted of two polydimethylsiloxane (PDMS) layers, a porous membrane, and a cover glass (Figure 2A,B). As reported previously [21], the mold was prepared by attaching 1.0 mm × 1.0 mm polystyrene strips to an acrylic plate. PDMS prepolymer (Silpot 184; Dow Toray, Tokyo, Japan) was meticulously deposited into the mold and cured at 90 °C. Inlet ports 2 mm in diameter were then formed in the cured PDMS sheet using a biopsy punch. An ipCELLCULTURE polyester track-etched membrane (pore size 1.0 um, it4ip, Louvain-la-Neuve, Belgium) was positioned between the two PDMS sheets, and the assembly was permanently bonded by oxygen plasma treatment for 20 s in a plasma reactor (PDC200; Yamato Scientific, Tokyo, Japan). To sterilize the device, 70% methanol was introduced into the channels, after which the device was exposed to UV light for 24 h. This treatment allowed complete evaporation of the methanol and sterilized the device before cell culture. Pipette tips were subsequently inserted into the inlet openings of all channels and used as medium reservoirs.

#### 2.2.2. Microdevice for Three-Dimensional (3D) Culture

The mold for the 3D culture microdevice (Figure 2C,D) was produced by SU-8-based soft lithography as previously described [21]. Briefly, SU-8 3025 photoresist (Kayaku Advanced Materials, Westborough, MA, USA) was spin-coated onto a glass slide, and a 500 µm high mold was generated by photolithography with a photomask. A PDMS sheet was then fabricated from this mold in the same manner as for the 2D culture device. Inlet holes 2 mm in diameter were punched at both ends of the channel, and the PDMS sheet was bonded to a cover glass by plasma treatment.

### 2.3. Cells and Medium

HepG2 cells, a human hepatoblastoma-derived cell line (RCB1648, Riken BRC, Ibaraki, Japan), were maintained in high-glucose Dulbecco modified Eagle medium (FUJIFILM Wako Pure Chemical, Osaka, Japan) with 1% minimum essential medium non-essential amino acid solution (FUJIFILM Wako Pure Chemical), 1% penicillin–streptomycin–amphotericin B suspension (FUJIFILM Wako Pure Chemical), and 10% fetal bovine serum (FBS; Biosera, Nuaille, France).

Immortalized human hepatic sinusoidal endothelial cells (TMNK-1; JCRB1564, JCRB, Osaka, Japan) were cultured with a 1:1 mixture of MCDB131 medium (Thermo Fisher Scientific, Waltham, MA, USA), supplemented with 1.25% L-glutamine solution (FUJIFILM Wako Pure Chemical), 1% penicillin–streptomycin–amphotericin B suspension, and 10% FBS, and endothelial cell growth medium (Promo Cell, Heidelberg, Germany) supplemented with 1% penicillin–streptomycin–amphotericin B.

### 2.4. Cell Culture

#### 2.4.1. Cell Culture in a 96-Well Plate

After washing with PBS, cells were detached using 0.25% trypsin–ethylenediaminetetraacetic acid (FUJIFILM Wako Pure Chemical) at 37 °C for 3 min. The harvested cells were resuspended in fresh medium and centrifuged. The resulting pellet was resuspended with the medium, and the cells were cultured in a well at 37 °C for 24 h. Based on the bottom surface area of each well (0.32 cm^2^), the seeding density of 2.5 × 10^5^ cells/cm^2^ was calculated to correspond to approximately 8.0 × 10^4^ cells/well.

#### 2.4.2. Cell Culture in a Microfluidic Device

For preparation of the 2D culture microdevice, Cellmatrix Type I-C collagen solution (Nitta Gelatin, Osaka, Japan), diluted 10-fold with 1 mM HCl, was introduced into the cell culture chamber and left for more than 1 h to coat the porous membrane. The collagen solution was then removed, and the chamber was washed with PBS. A cell suspension adjusted to 1.9 × 10^5^ cells/cm^2^ was added, and the device was incubated for 2 h to allow the cells to attach to the porous membrane. Thereafter, 200 µL of culture medium was added to each channel, and the cells were maintained at 37 °C under 5% CO_2_. Because the cell culture area of the device was 0.1 cm^2^, this seeding density was equivalent to approximately 1.9 × 10^4^ cells per device.

For the 3D culture microdevice, cells adjusted to 3.0 × 10^7^ cells/mL were suspended in a hydrogel consisting of 25 mg/mL bovine plasma-derived fibrinogen (Sigma-Aldrich, St. Louis, MO, USA) and thrombin (Sigma-Aldrich) prepared in saline at the prescribed concentration. This cell–hydrogel mixture was loaded into the gel channel with a micropipette. The device was then incubated at 37 °C for 15 min to promote gelation. Afterward, 200 µL of culture medium was added to each adjacent medium channel, and the cells were cultured at 37 °C in 5% CO_2_. As the gel region had a volume of 10 µL, the total number of cells introduced into each device was approximately 3.0 × 10^5^.

### 2.5. Cell Viability Assessment

#### 2.5.1. Cell Viability Assay

Viability of preserved cells was evaluated using the Cell Counting Kit-8 (CCK-8; Dojindo, Kumamoto, Japan) as previously described [20]. In the 2D culture MPS, the medium reservoirs were removed, the medium or preservation solution was aspirated, and the cells were rinsed with fresh medium. Next, 20 µL of medium containing CCK-8 reagent at a 10:1 ratio was added to the device, followed by incubation at 37 °C for 30 min. The reaction solution was collected, diluted 10-fold with PBS, and transferred to a 96-well plate. Absorbance at 450 nm was then measured using a 2300 EnSpire Multilabel Plate Reader (Perkin Elmer, Waltham, MA, USA). All data are presented as average ± standard deviation in the graphs and in Supplementary Tables S1–S6.

For the 3D culture MPS, the same basic procedure was used except that the CCK-8-containing medium was introduced into two medium channels (20 µL each) and incubated for 2 h before collection and dilution. Because the initial seeding density was identical across experiments, the measured absorbance values were used directly as an index of relative cell viability. Viability was calculated by normalizing the absorbance of preserved samples to that of non-preserved controls. In some instances, values above 100% were obtained; these were considered to reflect differences in metabolic activity and assay sensitivity rather than a true increase in cell number. Live/dead staining was additionally performed to complement the metabolic activity-based assay.

#### 2.5.2. Live/Dead Staining

Cell viability was also examined by fluorescent live/dead staining using Calcein AM (green; Dojindo) to label live cells and propidium iodide (PI; red; Sigma-Aldrich) to label dead cells. In the 2D culture MPS, the culture medium or preservation solution was aspirated, after which the cells were washed with fresh medium, and the wash solution was removed. A total of 100 µL of staining solution (1 mg/mL Calcein AM:1 mg/mL PI:PBS = 1:1:498) was then applied, followed by incubation at 37 °C for 20 min.

Cells in the 3D culture MPS were washed in the same manner. Subsequently, 100 µL of staining solution (1 mg/mL Calcein AM:1 mg/mL PI:PBS = 2:1:997) was introduced into two adjacent medium channels and incubated at 37 °C for 2 h. Fluorescence images were obtained using an inverted fluorescence microscope (IX71; Olympus, Tokyo, Japan).

#### 2.5.3. Phalloidin Staining

To assess cell morphology, cytoskeletal staining was carried out. Cells were fixed with 4% paraformaldehyde in phosphate buffer (FUJIFILM Wako Pure Chemical) for 30 min at room temperature and then permeabilized with 0.5% Triton X-100 (Sigma-Aldrich) in PBS for 30 min at room temperature. For the 2D culture MPS, Phalloidin-iFluor 555 solution (1 mg/mL; Cayman Chemical, Ann Arbor, MI, USA) was diluted 1000-fold in PBS, and 50 µL of the staining solution was applied, followed by 20 min incubation. For the 3D culture MPS, the dye was diluted 333-fold, and 50 µL of the solution was introduced into each adjacent medium channel, followed by 1 h incubation. After staining, the cells were observed with either a confocal laser scanning microscope (FV-300/IX71; Olympus) or a fluorescence microscope (BZ-9000; Keyence, Osaka, Japan).

## 3. Results

### 3.1. Optimization of Preservation Conditions for TMNK-1 Cells Cultured in a Well Plate

To investigate preservation conditions for endothelial cells, TMNK-1 cells were cultured in well plates and subjected to supercooling preservation. The effects of cooling rate, rewarming rate, preservation solutions, and preservation temperature on cell viability and morphology were evaluated.

#### 3.1.1. Cooling Rate

**Figure 3:**
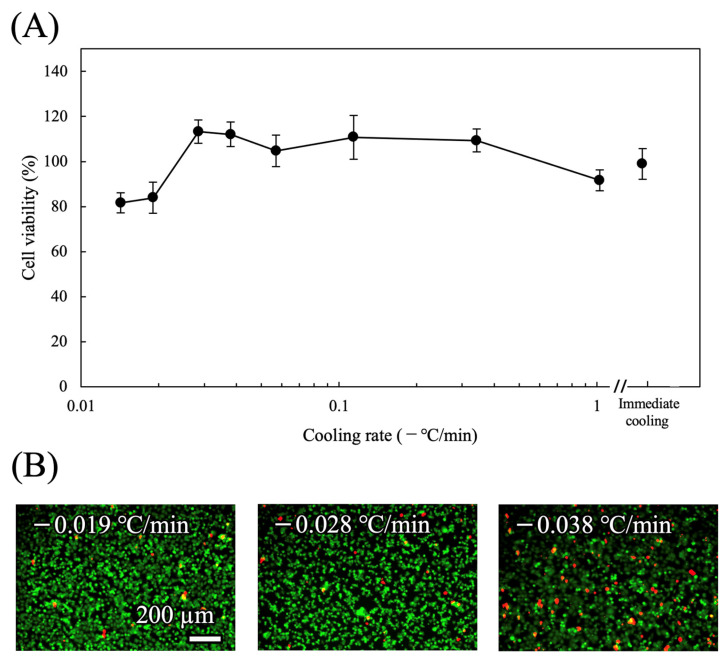
Effect of cooling rate on cell viability. (**A**) Viability of TMNK-1 cells following preservation at −4 °C for 24 h under various cooling rates (n = 5). The rewarming rate was fixed at +1.0 °C/min. (**B**) Representative live/dead fluorescence images of cells after preservation under the indicated cooling rates. Live cells are shown in green and dead cells in red.

The effects of cooling rate on cell viability were assessed (Figure 3A). Cells were cooled from 37 °C to −4 °C at different cooling rates and preserved for 24 h, followed by rewarming to 37 °C at +1.0 °C/min. Unlike HepG2 cells [20], cell viability remained at comparable levels (approximately 100%) across all tested cooling rates, including immediate cooling and −0.028 °C/min (optimal condition for HepG2). Representative live/dead images (Figure 3B) showed predominantly live cells under all conditions, with no apparent differences in cell morphology among the cooling rates tested. Based on these results, two conditions—immediate cooling and −0.028 °C/min—were selected for subsequent evaluation of rewarming rates.

#### 3.1.2. Rewarming Rate

**Figure 4:**
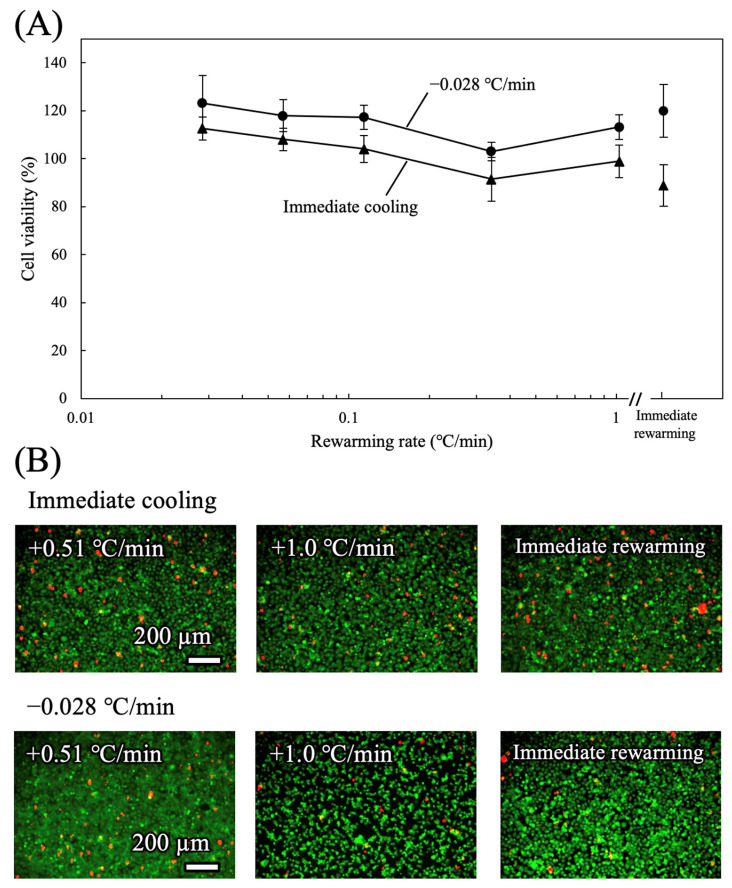
Effect of rewarming rate on cell viability. (**A**) Viability of TMNK-1 cells following preservation at −4 °C for 24 h under various rewarming rates (n = 5). The cooling condition was either immediate cooling or controlled cooling at −0.028 °C/min. (**B**) Representative live/dead fluorescence images of cells after preservation under the indicated rewarming rates. Live cells are shown in green and dead cells in red.

The effects of rewarming rate on cell viability were assessed (Figure 4A). Cells were cooled from 37 °C to −4 °C either immediately or at −0.028 °C/min and preserved for 24 h, followed by rewarming to 37 °C at different rates. High viability was maintained across all tested rewarming rates. However, cells cooled at a rate of −0.028 °C/min exhibited slightly higher viability than those with immediate cooling. Therefore, the cooling rate for TMNK-1 was set to −0.028 °C/min. To facilitate future co-culture of HepG2 and TMNK-1 cells, the rewarming rate was set at +1.0 °C/min, which was optimal for HepG2 cells in a previous study [20]. Representative live/dead images (Figure 4B) showed that most cells remained viable under all conditions tested. Based on these observations, a cooling rate of −0.028 °C/min and a rewarming rate of +1.0 °C/min were used in subsequent experiments.

#### 3.1.3. Preservation Solution

**Figure 5:**
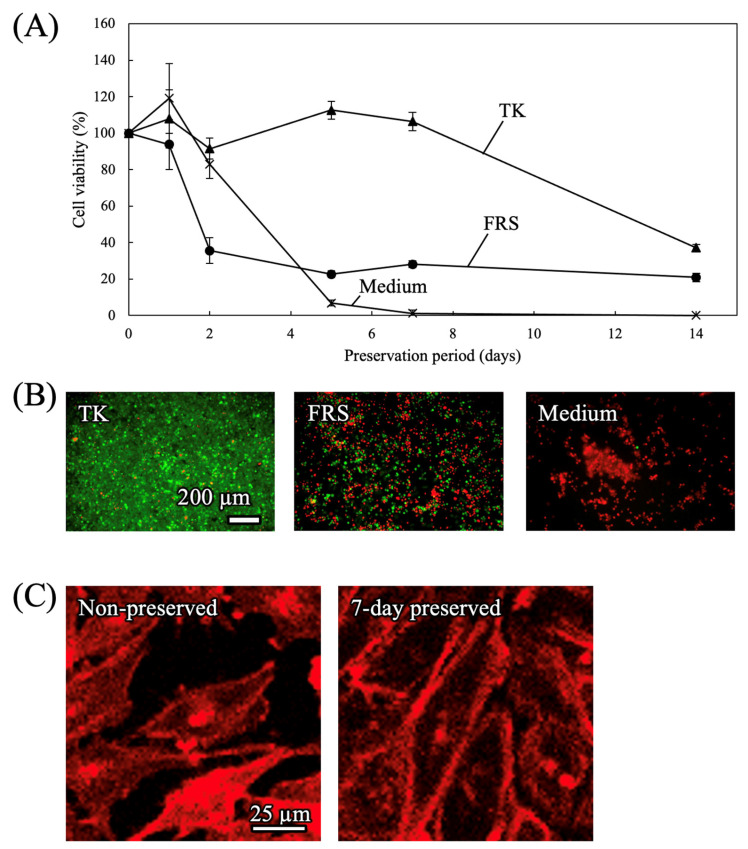
Viability of TMNK-1 cells after mid-term supercooling preservation at −4 °C. Cooling rate: −0.028 °C/min and rewarming rate: +1.0 °C/min. (**A**) Time course of cell viability preserved in different preservation solutions (n = 5). (**B**) Live/dead images obtained after supercooling preservation for 7 days in different preservation solutions. Live cells are shown in green and dead cells in red. (**C**) Cytoskeletal images of non-preserved and 7-day-preserved cells in TK obtained by phalloidin staining.

The effect of the preservation solution on cell viability was assessed (Figure 5A). Cells were preserved at −4 °C using each of the different preservation solutions (TK and FRS) and medium. Cells preserved with TK showed higher viability compared to other solutions. Representative live/dead images after a 7-day preservation are illustrated in Figure 5B. These findings indicate that TK was suitable for preserving TMNK-1. Phalloidin staining (Figure 5C) indicated that cytoskeletal structures were observable after 7 days of preservation, with no apparent morphological disruption compared to non-preserved cells.

#### 3.1.4. Comparison with Different Preservation Conditions

**Figure 6:**
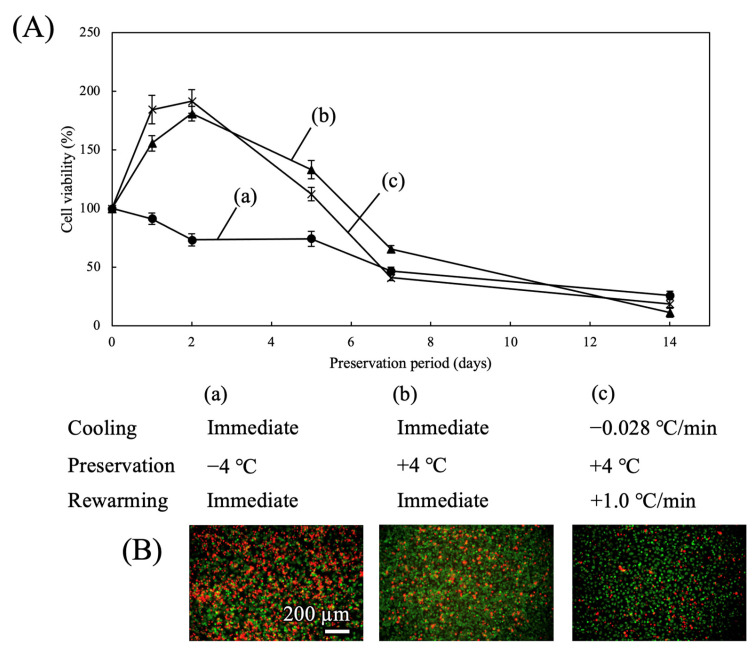
Viability of TMNK-1 cells after mid-term preservation in TK under different conditions. (**A**) Time course of cell viability (n = 5). (**B**) Live/dead images obtained after preservation for 7 days under different conditions. Live cells are shown in green and dead cells in red.

The effect of preservation temperature was evaluated by comparing −4 °C and +4 °C conditions (Figure 6A). Cell viability at +4 °C remained relatively high up to 5 days. After 7 days, however, viability reduced to 40–60%. Representative live/dead images of cells preserved for 7 days at +4 °C are illustrated in Figure 6B. Unlike cells preserved under optimal conditions, a high proportion of dead cells were observed after 7 days of preservation at +4 °C. In contrast, supercooling preservation at −4 °C (cooling rate of −0.028 °C/min and rewarming rate of +1.0 °C/min) maintained high TMNK-1 cell viability for up to 7 days when TK was used as the preservation solution (Figure 5A).

### 3.2. Optimization of Preservation Conditions for MPS

#### 3.2.1. Preservation of Cells in 2D Culture MPS

Cells grown on a porous membrane in the 2D microfluidic device were preserved at −4 °C. HepG2 and TMNK-1 cells were seeded onto the membrane at 1.9 × 10^5^ cells/cm^2^, allowed to adhere for 2 h, and then precultured for 24 h. After the preculture period, the medium in the MPS was removed and replaced with FRS for HepG2 cells or TK for TMNK-1 cells. The devices were cooled to −4 °C at a rate of −0.028 °C/min. After the preservation step, they were rewarmed from −4 °C to 37 °C at +1.0 °C/min. The preservation solution was then removed, the cells were washed with fresh medium, and the culture was continued at 37 °C for another 24 h.

**Figure 7:**
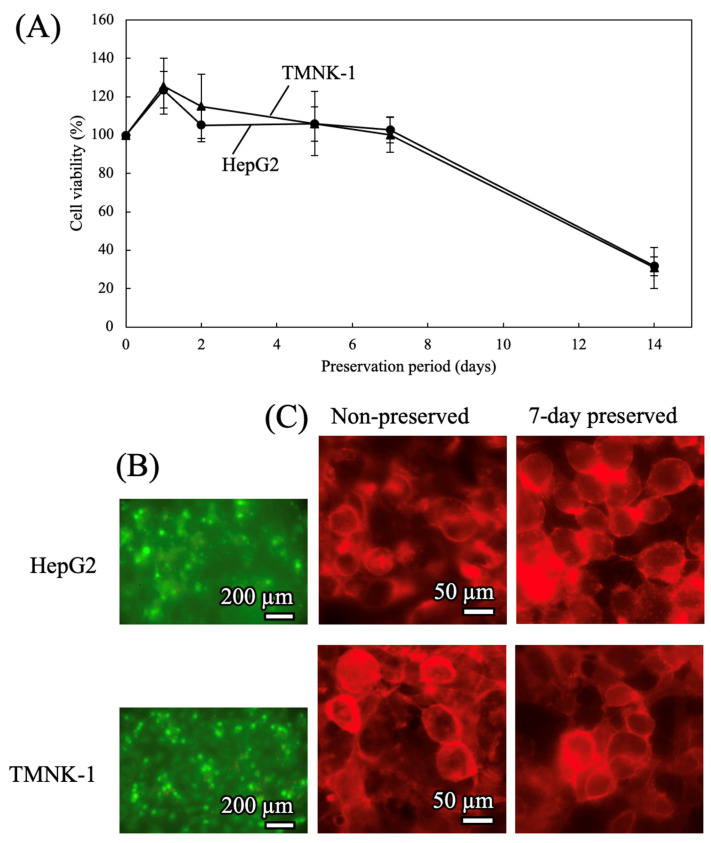
Cell viability after mid-term preservation in 2D culture microphysiological systems. Cells were preserved under a cooling rate of −0.028 °C/min and a rewarming rate of +1.0 °C/min. (**A**) Time course of viability during preservation (n = 5). (**B**) Representative live/dead fluorescence images after 7 days of preservation. Live cells are shown in green and dead cells in red. (**C**) Cytoskeletal structures of non-preserved and 7-day-preserved cells visualized by phalloidin staining.

The resulting post-preservation viability is shown in Figure 7A. Both HepG2 and TMNK-1 cells maintained high viability in the 2D MPS even after 7 days of preservation. Representative live/dead staining images of cells preserved for 7 days (Figure 7B and Supplementary Figure S1) show that most cells remained viable in both cell types. Likewise, representative phalloidin-stained images (Figure 7C) showed no detectable difference in cytoskeletal morphology between non-preserved cells and cells preserved for 7 days.

#### 3.2.2. Preservation of Cells in 3D Culture MPS

Cells embedded in a hydrogel and cultured in the 3D culture MPS were also preserved at −4 °C. HepG2 and TMNK-1 cells were suspended in fibrin gel at a density of 3.0 × 10^7^ cells/mL, and the cell-containing gel was introduced into the 3D microdevice. Culture medium was then added, and the cells were precultured for 24 h. After preculture, the medium was removed and replaced with FRS for HepG2 cells or TK for TMNK-1 cells. The devices were cooled, preserved, and then rewarmed under the same conditions as the 2D culture MPS described in Section 3.2.1. Subsequently, the preservation solution was removed, the cells were washed with fresh medium, and the cultures were maintained at 37 °C for a further 24 h.

**Figure 8:**
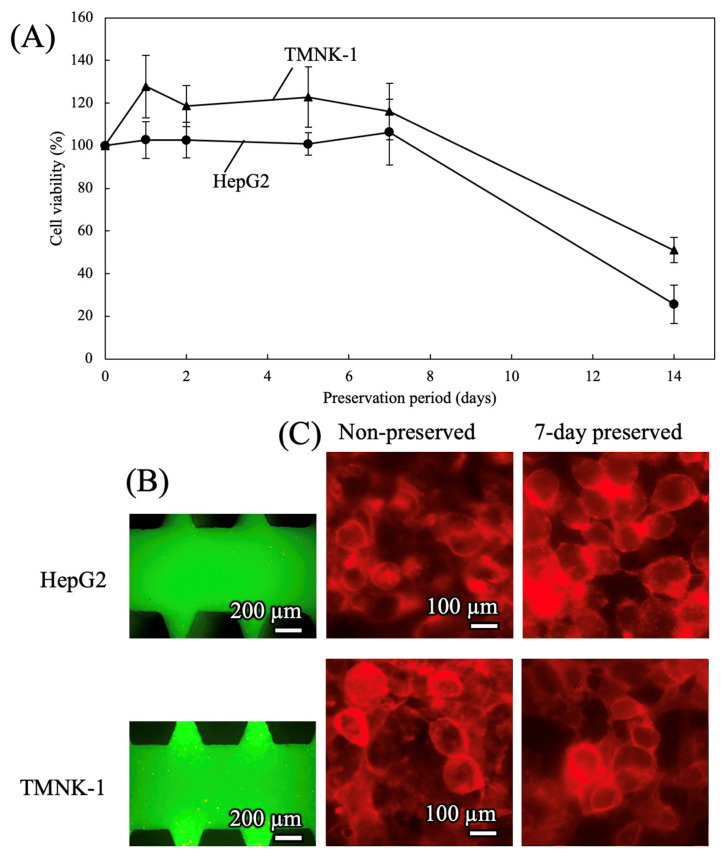
Cell viability after mid-term preservation in 3D culture microphysiological systems. Cells were preserved under a cooling rate of −0.028 °C/min and a rewarming rate of +1.0 °C/min. (**A**) Time course of viability during preservation (n = 5). (**B**) Representative live/dead fluorescence images after 7 days of preservation. Live cells are shown in green and dead cells in red. (**C**) Cytoskeletal structures of non-preserved and 7-day-preserved cells visualized by phalloidin staining.

Cell viability after preservation is presented in Figure 8A. In the 3D MPS, both HepG2 and TMNK-1 cells retained high viability even after 7 days of preservation. Representative live/dead staining images of the 7-day-preserved samples (Figure 8B and Supplementary Figure S2) indicate that most cells were alive in both cell types. Representative phalloidin-stained images (Figure 8C) further showed no apparent differences in cytoskeletal morphology between the non-preserved controls and the cells preserved for 7 days.

## 4. Discussion

In this study, a supercooling-based preservation method was investigated for adherently cultured endothelial cells and its applicability to MPS. The results showed that TMNK-1 cells maintained cell viability and observable cytoskeletal structures after preservation at −4 °C under the tested conditions. Similar trends were observed in both 2D and 3D culture MPS, suggesting that the conditions identified in well plate experiments can be applied to microdevice-based cultures within the scope of this study. Although a temperature-controlled CO_2_ incubator was used in this study, the protocol itself relies on controlled temperature changes and may be implemented using other systems capable of regulating cooling and rewarming rates. The preservation performance may be associated with reduced cellular activity at subzero temperatures without ice formation [18]. At −4 °C, metabolic activity is expected to be suppressed, which may limit the accumulation of cellular damage during storage. In contrast, at +4 °C, residual metabolic activity may lead to gradual nutrient depletion and accumulation of metabolic byproducts, potentially contributing to the decrease in cell viability observed during prolonged storage. The preservation solution also influenced outcomes, with TK showing higher cell viability compared to other conditions for preservation of TMNK-1 cells. Although TK is a commercially available solution and its detailed composition was not disclosed, its protective effect may be related to the mitigation of low-temperature stress, such as osmotic imbalance, membrane phase transitions, and oxidative stress. However, the underlying mechanisms remain unclear. Cooling and rewarming rates showed limited influence on TMNK-1 cell viability within the tested range. However, this observation is based on a single cell type and a 24 h preservation condition and therefore should be interpreted within this experimental scope. Subsequent research employing diverse cell types and varying storage periods may elucidate general trends. The applicability of this method to MPS was evaluated using basic systems, including membrane-based 2D and hydrogel-based 3D cultures, and cell viability and morphology were maintained. The applicability to more complex MPS platforms with multiple cell types or perfusion remains to be determined.

In this study, cell viability was evaluated using CCK-8 assays and fluorescence staining. As CCK-8 reflects metabolic activity, changes in metabolism during preservation may affect the measured viability, which may explain values exceeding 100%. Furthermore, given that the objective of this study was a preliminary investigation of the preservation conditions, no functional assessments were performed that were specific to each cell type. Consequently, the extent to which cellular function is maintained should be investigated in future studies. This study evaluated only two immortalized hepatic cells. However, differences in low-temperature sensitivity between immortalized and primary cells have been reported [19]. Therefore, the applicability of this method to other cell types, including primary cells, remains to be investigated. Furthermore, only a single preservation cycle was examined. In practical applications, repeated cooling and rewarming may be required, and the effects of multiple cycles on cell viability and function should be evaluated in future studies. Compared to conventional methods, supercooling enables storage at subzero temperatures without ice formation, which may reduce freezing-related damage while suppressing metabolism more effectively than hypothermic preservation [18]. However, direct comparisons with existing preservation methods were not performed, and the present findings should be considered as an initial evaluation. Overall, supercooling preservation at −4 °C enabled short- to mid-term storage of adherently cultured cells under the conditions tested in this study. Following further validation, including the evaluation of cell-specific functions and assessments using more complex MPS, this method may be put into practical use.

## 5. Conclusions

In this study, a supercooling preservation method was investigated for adherently cultured human cells in both well plate and MPS formats. Using TMNK-1 cells as a model, preservation conditions were evaluated, including cooling and rewarming rates and preservation solutions. Within the tested conditions, cell viability was maintained after preservation at −4 °C, and TK showed higher performance compared to other solutions. The optimized conditions were also applied to HepG2 and TMNK-1 cells cultured in 2D and 3D MPS models. Under these conditions, cell viability and morphology were maintained after up to 7 days of preservation. However, this study was conducted using a limited number of cell lines and relatively simple culture systems. Consequently, the broader applicability of this method to other cell types, more sophisticated MPS platforms, and functional outcomes remains to be elucidated.

## Data Availability

The raw and processed data required to reproduce these findings will be made available upon reasonable request to the corresponding author.

## Supplementary Materials

The following supporting information can be downloaded at https://www.mdpi.com/article/10.3390/cells15070619/s1, Table S1: Effects of cooling rate on cell viability; Table S2: Effects of rewarming rate on cell viability; Table S3: Viability of TMNK-1 cells after mid-term supercooling preservation at −4 °C; Table S4: Viability of TMNK-1 cells after mid-term preservation in TK under different conditions; Table S5: Viability after mid-term supercooling preservation of cells cultured in 2D culture microphysiological systems; Table S6: Viability after mid-term supercooling preservation of cells cultured in 3D culture microphysiological systems; Figure S1: Live/dead separate images obtained after supercooling preservation of cells cultured in 2D culture MPS for 7 days; Figure S2: Live/dead separate images obtained after supercooling preservation of cells cultured in 3D culture MPS for 7 days.

## Abbreviations

MPS	Microphysiological systems
DMSO	Dimethyl sulfoxide
FRS	HypoThermosol FRS
TK	Thelio Keep
PDMS	Polydimethylsiloxane
FBS	Fetal Bovine Serum
PBS	Phosphate-Buffered Saline
CCK-8	Cell Counting Kit-8
PI	Propidium Iodide
