# Supplementary material for: Development of Supercooling Preservation Method for Adherently Cultured Endothelial Cells and Its Application to Microphysiological Systems
Source: Cells. 2026 Mar 30;15(7):619. doi: 10.3390/cells15070619 (PMC13072380; doi:10.3390/cells15070619)
Supplement: Supplementary file 1 [file cells-15-00619-s001.zip › cells-4200766-supplementary.pdf]

**Supplementary Table S1.** Effects of cooling rate on cell viability. Absorbance and viability of TMNK-1 cells preserved at -4 °C for 24 h under different cooling rates (n=5, Average ± Standard deviation). Rewarming rate is fixed at +1.0 °C/min.

| Cooling rate (−°C/min) | Absorbance | | Cell viability (%) |
|---|---|---|---|
| | Cells without preservation | Cells after preservation | |
| 0.014 | 0.520 ± 0.012 | 0.425 ± 0.021 | 82 ± 4 |
| 0.019 | 0.583 ± 0.041 | 0.289 ± 0.021 | 84 ± 7 |
| 0.028 | 0.411 ± 0.013 | 0.466 ± 0.015 | 113 ± 5 |
| 0.038 | 0.552 ± 0.019 | 0.618 ± 0.021 | 112 ± 5 |
| 0.057 | 0.595 ± 0.021 | 0.623 ± 0.036 | 105 ± 7 |
| 0.11 | 0.521 ± 0.025 | 0.577 ± 0.043 | 111 ± 7 |
| 0.34 | 0.640 ± 0.019 | 0.700 ± 0.024 | 109 ± 5 |
| 1.0 | 0.482 ± 0.008 | 0.441 ± 0.021 | 92 ± 5 |
| Immediate | 0.482 ± 0.008 | 0.477 ± 0.032 | 99 ± 7 |

**Supplementary Table S2.** Effects of rewarming rate on cell viability. Absorbance and viability of TMNK-1 cells preserved at -4 °C for 24 h with different rewarming rates (n = 5, Average ± Standard deviation). Cooling rate is fixed at immediate cooling or -0.028 °C/min.

Cooling rate : Immediate

| Rewarming rate (°C/min) | Absorbance | | Cell viability (%) |
|---|---|---|---|
| | Cells without preservation | Cells after preservation | |
| 0.028 | 0.488 ± 0.012 | 0.549 ± 0.020 | 113 ± 5 |
| 0.057 | 0.483 ± 0.011 | 0.521 ± 0.019 | 108 ± 5 |
| 0.11 | 0.466 ± 0.021 | 0.485 ± 0.014 | 104 ± 6 |
| 0.34 | 0.530 ± 0.051 | 0.485 ± 0.013 | 91 ± 9 |
| 1.0 | 0.482 ± 0.008 | 0.477 ± 0.032 | 99 ± 7 |
| Immediate | 0.530 ± 0.051 | 0.471 ± 0.008 | 89 ± 9 |

Cooling rate : −0.028 °C/min

| Rewarming rate (°C/min) | Absorbance | | Cell viability (%) |
|---|---|---|---|
| | Cells without preservation | Cells after preservation | |
| 0.028 | 0.549 ± 0.043 | 0.676 ± 0.034 | 123 ± 12 |
| 0.057 | 0.385 ± 0.020 | 0.455 ± 0.011 | 118 ± 7 |
| 0.11 | 0.448 ± 0.012 | 0.525 ± 0.017 | 117 ± 5 |
| 0.34 | 0.568 ± 0.012 | 0.586 ± 0.019 | 103 ± 4 |
| 1.0 | 0.411 ± 0.013 | 0.466 ± 0.015 | 113 ± 5 |
| Immediate | 0.543 ± 0.045 | 0.652 ± 0.026 | 120 ± 11 |

**Supplementary Table S3.** Viability of TMNK-1 cells after mid-term supercooling preservation at -4 °C. Cooling rate: -0.028 °C/min and rewarming rate: +1.0 °C/min. Time course of absorbance and cell viability preserved in different preservation solutions (n = 5, Average ± Standard deviation).

Preservation solution : TK

| Preservation period (days) | Absorbance | | Cell viability (%) |
|---|---|---|---|
| | Cells without preservation | Cells after preservation | |
| 1 | 0.705 ± 0.018 | 0.760 ± 0.111 | 108 ± 16 |
| 2 | 0.705 ± 0.018 | 0.646 ± 0.037 | 92 ± 6 |
| 5 | 0.705 ± 0.018 | 0.794 ± 0.028 | 113 ± 5 |
| 7 | 0.253 ± 0.010 | 0.270 ± 0.007 | 106 ± 5 |
| 14 | 0.613 ± 0.021 | 0.228 ± 0.008 | 37 ± 2 |

Preservation solution : FRS

| Preservation period (days) | Absorbance | | Cell viability (%) |
|---|---|---|---|
| | Cells without preservation | Cells after preservation | |
| 1 | 0.705 ± 0.018 | 0.661 ± 0.095 | 94 ± 14 |
| 2 | 0.345 ± 0.026 | 0.123 ± 0.022 | 36 ± 7 |
| 5 | 0.572 ± 0.017 | 0.130 ± 0.010 | 23 ± 2 |
| 7 | 0.253 ± 0.010 | 0.071 ± 0.018 | 28 ± 2 |
| 14 | 0.613 ± 0.021 | 0.176 ± 0.010 | 21 ± 2 |

Preservation solution : Medium

| Preservation period (days) | Absorbance | | Cell viability (%) |
|---|---|---|---|
| | Cells without preservation | Cells after preservation | |
| 1 | 0.705 ± 0.018 | 0.840 ± 0.133 | 119 ± 19 |
| 2 | 0.705 ± 0.018 | 0.585 ± 0.053 | 83 ± 8 |
| 5 | 0.705 ± 0.018 | 0.048 ± 0.011 | 7 ± 2 |
| 7 | 0.705 ± 0.018 | 0.009 ± 0.012 | 1 ± 2 |
| 14 | 0.253 ± 0.010 | -0.005 ± 0.010 | 0 |

**Supplementary Table S4.** Viability of TMNK-1 cells after mid-term preservation in TK under different conditions. Time course of absorbance and cell viability (n = 5, Average ± Standard deviation).

Cooling rate : Immediate, Preservation temperature : −4 ℃, Rewarming rate : Immediate

| Preservation period (days) | Absorbance | | Cell viability (%) |
|---|---|---|---|
| | Cells without preservation | Cells after preservation | |
| 1 | 0.703 ± 0.025 | 0.641 ± 0.026 | 91 ± 5 |
| 2 | 0.976 ± 0.059 | 0.715 ± 0.025 | 73 ± 5 |
| 5 | 0.703 ± 0.025 | 0.522 ± 0.041 | 74 ± 9 |
| 7 | 0.703 ± 0.025 | 0.328 ± 0.019 | 47 ± 3 |
| 14 | 0.415 ± 0.047 | 0.108 ± 0.006 | 26 ± 3 |

Cooling rate : Immediate, Preservation temperature : ＋4 ℃, Rewarming rate : Immediate

| Preservation period (days) | Absorbance | | Cell viability (%) |
|---|---|---|---|
| | Cells without preservation | Cells after preservation | |
| 1 | 0.398 ± 0.012 | 0.620 ± 0.018 | 156 ± 7 |
| 2 | 0.384 ± 0.012 | 0.694 ± 0.009 | 181 ± 6 |
| 5 | 0.398 ± 0.012 | 0.530 ± 0.027 | 133 ± 8 |
| 7 | 0.470 ± 0.017 | 0.307 ± 0.009 | 65 ± 3 |
| 14 | 0.449 ± 0.023 | 0.051 ± 0.018 | 11 ± 4 |

Cooling rate : −0.028 ℃/min, Preservation temperature : ＋4 ℃, Rewarming rate : ＋1.0 ℃/min

| Preservation period (days) | Absorbance | | Cell viability (%) |
|---|---|---|---|
| | Cells without preservation | Cells after preservation | |
| 1 | 0.384 ± 0.012 | 0.707 ± 0.040 | 184 ± 12 |
| 2 | 0.384 ± 0.012 | 0.734 ± 0.014 | 191 ± 10 |
| 5 | 0.384 ± 0.012 | 0.431 ± 0.018 | 112 ± 6 |
| 7 | 0.395 ± 0.018 | 0.162 ± 0.006 | 41 ± 2 |
| 14 | 0.443 ± 0.025 | 0.082 ± 0.016 | 18 ± 4 |

**Supplementary Table S5.** Viability after mid-term supercooling preservation of cells cultured in 2D-culture microphysiological systems. Cooling rate: -0.028 ℃/min and rewarming rate: +1.0 ℃/min. Time course of absorbance and cell viability (n = 5, Average ± Standard deviation).

Cell : HepG2

| Preservation period (days) | Absorbance | | Cell viability (%) |
|---|---|---|---|
| | Cells without preservation | Cells after preservation | |
| 1 | 0.203 ± 0.014 | 0.251 ± 0.010 | 124 ± 10 |
| 2 | 0.203 ± 0.014 | 0.214 ± 0.010 | 105 ± 9 |
| 5 | 0.203 ± 0.014 | 0.215 ± 0.011 | 106 ± 9 |
| 7 | 0.203 ± 0.014 | 0.209 ± 0.011 | 103 ± 7 |
| 14 | 0.203 ± 0.014 | 0.064 ± 0.009 | 32 ± 5 |

Cell : TMNK-1

| Preservation period (days) | Absorbance | | Cell viability (%) |
|---|---|---|---|
| | Cells without preservation | Cells after preservation | |
| 1 | 0.126 ± 0.016 | 0.158 ± 0.015 | 125 ± 15 |
| 2 | 0.126 ± 0.016 | 0.144 ± 0.012 | 114 ± 10 |
| 5 | 0.126 ± 0.016 | 0.133 ± 0.012 | 106 ± 17 |
| 7 | 0.126 ± 0.016 | 0.126 ± 0.008 | 100 ± 9 |
| 14 | 0.126 ± 0.016 | 0.039 ± 0.013 | 31 ± 11 |

**Supplementary Table S6.** Viability after mid-term supercooling preservation of cells cultured in 3D-culture microphysiological systems. Cooling rate: -0.028 °C/min and rewarming rate: +1.0 °C/min. Time course of absorbance and cell viability (n = 5, Average ± Standard deviation).

Cell : HepG2

| Preservation period (days) | Absorbance | | Cell viability (%) |
|---|---|---|---|
| | Cells without preservation | Cells after preservation | |
| 1 | 0.126 ± 0.009 | 0.129 ± 0.011 | 103 ± 9 |
| 2 | 0.126 ± 0.009 | 0.129 ± 0.011 | 103 ± 9 |
| 5 | 0.126 ± 0.009 | 0.127 ± 0.007 | 101 ± 5 |
| 7 | 0.126 ± 0.009 | 0.133 ± 0.031 | 106 ± 15 |
| 14 | 0.126 ± 0.009 | 0.032 ± 0.011 | 26 ± 9 |

Cell : TMNK-1

| Preservation period (days) | Absorbance | | Cell viability (%) |
|---|---|---|---|
| | Cells without preservation | Cells after preservation | |
| 1 | 0.102 ± 0.010 | 0.130 ± 0.009 | 128 ± 15 |
| 2 | 0.102 ± 0.010 | 0.121 ± 0.010 | 119 ± 10 |
| 5 | 0.102 ± 0.010 | 0.125 ± 0.014 | 123 ± 14 |
| 7 | 0.102 ± 0.010 | 0.118 ± 0.013 | 116 ± 13 |
| 14 | 0.102 ± 0.010 | 0.052 ± 0.006 | 51 ± 6 |

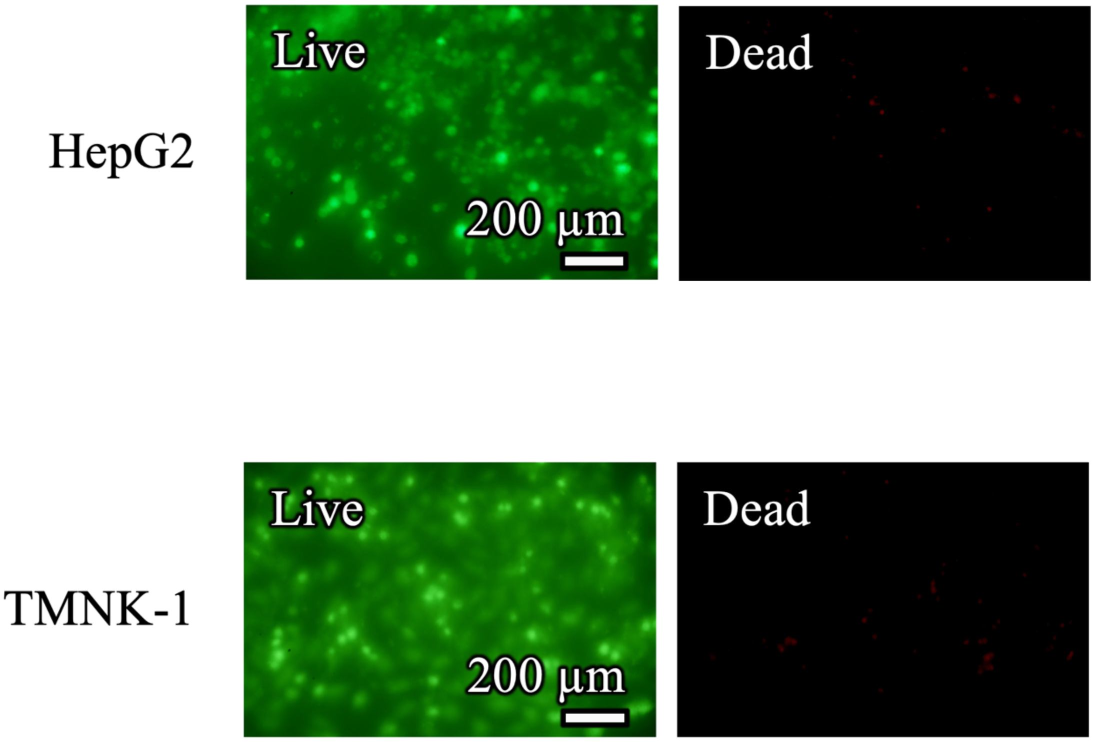


**Supplementary Figure S1.** Live/dead separate images obtained after supercooling preservation of cells cultured in 2D-culture MPS for 7 days. Cooling rate: -0.028 °C/min and rewarming rate: +1.0 °C/min.

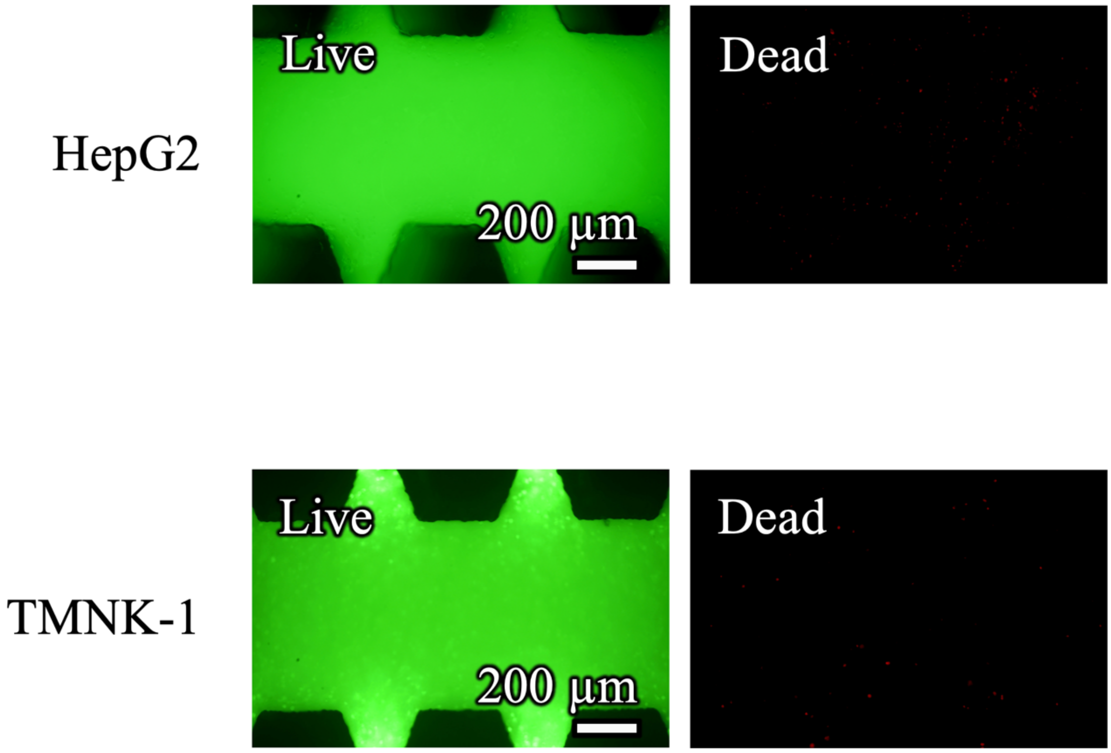


**Supplementary Figure S2.** Live/dead separate images obtained after supercooling preservation of cells cultured in 3D-culture MPS for 7 days. Cooling rate: -0.028 °C/min and rewarming rate: +1.0 °C/min.
